# Phase 2 study of futibatinib in combination with pembrolizumab in patients with FGF19 expressing advanced hepatocellular carcinoma

**DOI:** 10.1093/oncolo/oyag343

**Published:** 2026-08-25

**Authors:** Nguyen H Tran, Mathias E Palmer, Angela Ulrich, Shaylene A McCue, Amit Mahipal, Wen Wee Ma, Hani M Babiker, Umair Majeed, Thorvardur R Halfdanarson, Robert R McWilliams, Zhaohui Jin, Ryan Carr, Lionel Aurelien Kankeu Fonkoua, Leslie Washburn, Caitlin Conboy, Rondell P Graham, Michael Torbenson, Patrick Starlinger, Krishan R Jethwa, Christopher Hallemeier, Alexander Revzin, Scott M Thompson, Ajit H Goenka, Sudhakar K Venkatesh, Mohamad B Sonbol, Mitesh J Borad, Tanios Bekaii-Saab, Gregory J Gores, Lewis R Roberts, Julie K Heimbach, Fang-Shu Ou

**Affiliations:** Department of Oncology, Mayo Clinic, Rochester, MN 55905, United States; Department of Oncology, Mayo Clinic, Rochester, MN 55905, United States; Division of Clinical Trials and Biostatistics, Mayo Clinic, Rochester, MN 55905, United States; Division of Clinical Trials and Biostatistics, Mayo Clinic, Rochester, MN 55905, United States; Department of Oncology, University Hospitals Seidman Cancer Center and Case Western Reserve University, Cleveland, OH 44106, United States; Department of Hematology/Oncology, Cleveland Clinic Cancer Center, Cleveland, OH 44195, United States; Division of Hematology/Oncology, Mayo Clinic, Jacksonville, FL 32224, United States; Division of Hematology/Oncology, Mayo Clinic, Jacksonville, FL 32224, United States; Department of Oncology, Mayo Clinic, Rochester, MN 55905, United States; OSF HealthCare Cancer Institute, Peoria, IL 61603, United States; Department of Oncology, Mayo Clinic, Rochester, MN 55905, United States; Department of Oncology, Mayo Clinic, Rochester, MN 55905, United States; Department of Oncology, Mayo Clinic, Rochester, MN 55905, United States; Department of Oncology, Mayo Clinic, Rochester, MN 55905, United States; Department of Oncology, Mayo Clinic, Rochester, MN 55905, United States; Department of Laboratory Medicine and Pathology, Mayo Clinic, Rochester, MN 55905, United States; Department of Laboratory Medicine and Pathology, Mayo Clinic, Rochester, MN 55905, United States; Department of Surgery, Mayo Clinic, Rochester, MN 55905, United States; Department of Radiation Oncology, Mayo Clinic, Rochester, MN 55905, United States; Department of Radiation Oncology, Mayo Clinic, Rochester, MN 55905, United States; Department of Physiology and Biomedical Engineering, Mayo Clinic, Rochester, MN 55905, United States; Department of Radiology, Mayo Clinic, Rochester, MN 55905, United States; Department of Radiology, Mayo Clinic, Rochester, MN 55905, United States; Department of Radiology, Mayo Clinic, Rochester, MN 55905, United States; Division of Hematology/Oncology, Mayo Clinic, Phoenix, AZ 85054, United States; Division of Hematology/Oncology, Mayo Clinic, Phoenix, AZ 85054, United States; Division of Hematology/Oncology, Mayo Clinic, Phoenix, AZ 85054, United States; Division of Gastroenterology, Mayo Clinic, Rochester, MN 55905, United States; Division of Gastroenterology, Mayo Clinic, Rochester, MN 55905, United States; Department of Surgery and Transplant, Mayo Clinic, Rochester, MN 55905, United States; Division of Clinical Trials and Biostatistics, Mayo Clinic, Rochester, MN 55905, United States

**Keywords:** advanced hepatocellular carcinoma, futibatinib, pembrolizumab, FGFR inhibitor, immunotherapy

## Abstract

**Introduction:**

Optimal treatment following first-line immunotherapy for advanced hepatocellular carcinoma (HCC) remains uncertain. This study evaluated futibatinib, a pan-FGFR inhibitor, combined with pembrolizumab in previously treated patients with FGF19-expressing HCC.

**Methods:**

Eligible patients were ≥18 years old with advanced HCC, prior systemic therapy, Child-Pugh A-B7 liver function, ECOG 0-2, and FGF19 overexpression. Patients received futibatinib 20 mg orally daily plus pembrolizumab 200 mg intravenously every 3 weeks. The primary endpoint was progression-free survival at 6 months (PFS6). Secondary endpoints included overall survival (OS), objective response rate (ORR), quality of life, and safety.

**Results:**

Between August 2021 and March 2024, 14 patients enrolled and 13 were evaluable. Median age was 72 years (range, 60-87), 53.8% were male, 92.3% had Child-Pugh A liver function, and all had received prior immunotherapy. Median progression-free survival was 15 weeks (95% CI, 9.0-NE), with a PFS6 rate of 23.1% (95% CI, 8.6-62.3%). ORR was 0% (95% CI, 0-24.7%), while stable disease was observed in 53.9% of patients. Median OS was 55.7 weeks (95% CI, 47.3-NE). Grade ≥3 adverse events occurred in 69.2% of patients, and grade ≥4 adverse events in 15.4%. One grade 5 event (acute-on-chronic kidney injury) was deemed unrelated to treatment. Common adverse events included hyperphosphatemia, fatigue, diarrhea, elevated transaminases, dry eye, nail changes, rash, and oral mucositis.

**Conclusions:**

Futibatinib plus pembrolizumab demonstrated an acceptable safety profile but did not meet efficacy thresholds supporting further study in FGF19-expressing HCC. Effective post-immunotherapy treatment strategies remain an unmet need.

Implications for practice This study addresses a critical unmet need in advanced hepatocellular carcinoma by prospectively evaluating a biomarker-selected targeted therapy and immunotherapy combination in patients who had progressed after prior systemic treatment. Although the regimen did not demonstrate sufficient efficacy to warrant further development, the findings provide important evidence regarding the limitations of targeting the FGF19/FGFR pathway in combination with PD-1 blockade in this setting. Negative prospective studies are essential to advancing the field, as they help refine biomarker strategies, inform future trial design, and prevent the allocation of resources to ineffective approaches. These results contribute to improving treatment sequencing and therapeutic development for patients with advanced HCC.

## Introduction

Hepatocellular carcinoma (HCC) is the most prevalent form of primary liver cancer and ranks as the sixth most common malignancy worldwide, as well as the third leading cause of cancer-related mortality.^1^ While advances in first-line treatments for advanced HCC have improved overall survival,^2–4^ subsequent treatments are not well-established following currently approved first-line immunotherapy.

Fibroblast growth factor (FGF) and fibroblast growth factor receptor (FGFR) signaling is involved in broad biological processes including embryogenesis, angiogenesis, tissue homeostasis and cancer progression.^5^ In HCC, FGF-19 acts as a potential oncogenic driver through selective activation of FGFR4 with the co-receptor β-klotho, leading to downstream Ras-MAPK and PI3K-Akt signaling.^6–10^ FGF-19 overexpression arises primarily from amplification of the FGF19/CCND1 locus on 11q13 and from epigenetic alterations (approximately 25%).^7^^,^^11–13^ Experimental models show that aberrant FGF-19 promotes hepatocyte proliferation, dysplasia, tumor formation, and AFP upregulation, while FGF-19 or FGFR4 inhibition suppresses proliferation, invasion, and induces apoptosis.^14^^,^^15^ Clinically, FGF-19 amplification is associated with poor prognostic factors and worse outcomes, including higher recurrence risk and shorter survival.^11^^,^^16^^,^^17^ Currently, treatment options targeting FGF-19/FGFR4 pathway are not well established.^18^

Futibatinib is a novel and highly selective irreversible pan-FGFR inhibitor. In the phase I dose-escalation study, 86 patients with solid tumor harboring FGF amplifications or FGFR aberrations showed manageable toxicity and had reasonable clinical activity.^19^ The recommended phase 2 dose was 20 mg daily. The most frequently reported treatment-related adverse events include hyperphosphatemia, diarrhea, dry mouth, nausea, and stomatitis.

FGFR-altered cancers have been correlated with non-T-cell-inflamed microenvironment.^20^ Palakurthi and colleagues confirmed this finding in genetically engineered mice showing significant decrease in T and natural killer cells, increased regulatory T cells, and exhaustion marker-positive T cells.^21^ The combination of erdafitinib with anti-PD-1 leads to decrease infiltration of immunosuppressive TAMs, increased NK and B cells and higher proliferative, activated T and NK cells.

Furthermore, the combination resulted in significant increase in both T-cell fraction and clonality resulting in improved survival. Thus, combining FGFR inhibitor with immunotherapy appeared synergistic.

Here, we present the results from the phase 2, single-arm study conducted at the Mayo Clinic in assessing the efficacy and safety of the combination futibatinib plus pembrolizumab in patients with FGF19 biomarker enriched following first line immunotherapy.

## Methods

### Patients

Eligible patients were: (1) at least 18 years old with advanced HCC (histologically confirmed or radiologically confirmed); (2) Barcelona Clinic Liver Cancer (BCLC) stage A-C and not suitable for a potentially curative treatment approach; (3) having received at least one line of systemic therapy, including prior checkpoint inhibitor. Additional inclusion criteria included at least one measurable lesion based on RECIST v1.1, using computed tomography (CT) or magnetic resonance imaging (MRI); Eastern Cooperative Oncology Group (ECOG) performance status (PS) of 0-2 and life expectancy of at least 12 weeks; Child-Pugh (CP) score ≤ B7and adequate organ function (liver, kidney, bone marrow). Significant cardiovascular impairment and autoimmune disease history (including, but not limited to, systemic lupus erythematosus, rheumatoid arthritis, inflammatory bowel disease, vascular thrombosis associated with antiphospholipid syndrome, Wegener’s granulomatosis, Sjögren’s syndrome, Guillain-Barré syndrome, multiple sclerosis, autoimmune thyroid disease, vasculitis, or glomerulonephritis) was not permitted.

### Study design

This was a single-arm, two-stage, phase II trial (NCT04828486) assessing the efficacy of futibatinib plus pembrolizumab in advanced HCC for patients with FGF19-expression after receiving at least one line of therapy.

FGF19 positivity was initially an eligibility criterion for the first 11 patients; however, due to slow accrual, the protocol was amended to allow enrollment prior to FGF19 testing. FGF19-expression was subsequently assessed post-enrollment, with positivity defined as ≥1% of tumor cells positive by immunohistochemistry (IHC) or in-situ hybridization (ISH). Patients received futibatinib 20 mg orally once daily on Days 1-21 and pembrolizumab 200 mg intravenously on Day 1 of each 21-day cycle, continuing treatment until disease progression or unacceptable toxicity.

The protocol was approved by the Mayo Clinic institutional review board (20-007235). The trial was conducted in accordance with the Good Clinical Practice guidelines of the International Council for Harmonization and the principles of the Declaration of Helsinki. All the patients provided written informed consent before enrollment. The trial was regularly monitored by the Mayo Clinic data and safety monitoring board. Data were collected by the Mayo Clinic, reviewed by the trial chairperson (the first author), and analyzed by all the authors. The authors wrote the manuscript and vouched for the accuracy and completeness of the data and for the fidelity of the trial to the protocol. No one who is not an author contributed to the writing of the manuscript.

### Endpoints

The primary endpoint was progression-free survival (PFS) at 6 months (PFS6) after registration. PFS was defined as the time from registration to the first documented disease progression, per RECIST v1.1 or death. Six months was defined as 27 weeks. Patients who did not experience disease progression or death were censored at their last disease evaluation date. Secondary endpoints included objective response (ORR), overall survival (defined as the time from registration to death due to any cause), adverse events and quality of life outcomes (measured by European Organization for Research and Treatment of Cancer Quality of Life Questionnaire [EORTC-QLQ-C30]). ORR was defined as the number of evaluable patients achieving a response (partial response [PR] or complete response [CR] per RECIST v1.1) during protocol treatment divided by the total number of evaluable patients. EORTC-QLQ-C30 raw score was calculated per scoring manual and transformed to scores ranging from 0 to 100 with higher score indicating better quality of life. Change in scores between baseline and first re-staging was calculated for each individual.

### IHC/ISH assay

IHC and RNA-ISH staining was performed at the Pathology Research Core (Mayo Clinic, Rochester, MN) using the Leica Bond RX stainer (Leica). Archived FFPE tissues were sectioned at 5 microns for either IHC or RNA-ISH staining. All samples were obtained prior to starting trial treatment.

Slides for FGF19-expression (IHC stain) were retrieved for 10 minutes using Epitope Retrieval 2 (EDTA; Leica), incubated in Protein Block (Dako) for 5 minutes, followed by FGF19 primary antibody (Rb. Polyclonal, Sigma HPA036082) for 15 minutes diluted to 1:300 in Background Reducing Diluent (Dako). The detection system used was Polymer Refine Detection System (Leica). Once the immunochemistry process was completed, slides were removed from stainer, rinsed in tap water for 5 minutes, dehydrated in increasing concentrations of ethyl alcohol, and cleared in 3 changes of xylene prior to permanent coverslipping in xylene-based medium.

Slides for FGF19-expression (RNA-ISH stain) were baked at 60 °C for one hour prior to loading in autostainer. Slides were baked again and dewaxed prior to been exposed to the retrieval solution, Epitope Retrieval 2 (EDTA; Leica) for 30 minutes at 95 °C and the Protease III pretreatment (ACD) for 15 minutes at 40 °C. The slides were incubated for 2 hours at 40 °C with either the Hs-PPIB 2.5 LS probe (positive control probe, ACD), HS-FGF19 2.5 LS (FGF19 probe, ACD) or DapB (negative control probe, ACD).

RNAscope 2.5 LS Reagent Kit-Brown (ACD) was used for the amplification steps, followed by visualization using DAB from 10 minutes, and counterstain with hematoxylin for 5 minutes and bluing (Bluing 2.5 LS reagent, ACD). Slides were removed from the stainer, rinsed in tap water, dehydrated in increasing concentrations of ethyl alcohol, and cleared in 3 changes of xylene prior to permanent coverslipping in xylene-based medium.

More than 1% of tumor cells positive by either method was considered positive.

### Assessment

Tumor response was assessed using CT/MRI scans, per RECIST v1.1, at screening and then every 9 weeks. Safety assessments consisted of monitoring and recording adverse events (AEs) of all severity grades, AEs were graded according to the Common Terminology Criteria for Adverse Events (CTCAE) of the National Cancer Institute version 5.0 from the time of informed consent until 30 days after the last dose or until the patient initiated a new anticancer therapy, whichever was earlier.

Ophthalmological assessments, including visual acuity and direct/indirect ophthalmoscopy, were performed at screening/baseline and at 4-6 weeks after the first dose of futibatinib. If patients reported symptoms of visual disturbances, these were to be managed according to clinical guidelines in conjunction with specialist consultation.

### Statistical analysis

The study planned to accrue 22 evaluable patients. Evaluable patients were defined as those who are eligible, consented, received any protocol treatment, without major violation, and positive for FGF19. With 22 evaluable patients, we had 80% power to detect PFS6 from 25% (historical control)^22^ to 47%, assuming a 1-sided significance level of 0.1. The trial had a single interim analysis for futility which was to be conducted after the 11th evaluable patient was enrolled and followed for 6 months. The study enrollment was to be closed if PFS6 was ≤30% during interim analysis.

The interim analysis was conducted and yielded a PFS6 of 27.3%, which was less than the decision boundary of 30%. As a result, the study did not meet the interim criteria to continue enrollment. Contemporaneously, due to slow enrollment, the sponsor also decided to permanently close accrual. For the remainder of this manuscript, we report the final analysis results using all patients enrolled.

Baseline characteristics are summarized by median (range) for continuous variables and count with percentage for categorical variables. EORTC-QLQ-C30 scores and follow up durations are summarized by median (range or IQR). The median PFS/OS and corresponding 95% confidence intervals, including PFS6 rate, were estimated using Kaplan-Meier method. Confidence intervals (CI) for binary estimates, such as ORR, were calculated according to the method of Clopper-Pearson.

## Results

### Baseline characteristics

Between August 17, 2021 and March 18, 2024, 45 patients were screened, 20 with IHC positive, and 14 enrolled. One patient withdrew consent before beginning protocol treatment. All 13 remaining patients had FGF19-expression (Figure S1). Among 13 evaluable patients, median age was 72 (range: 60-87) years, 7 (53.8%) were male, 12 with CP A (92.3%), 7 with BCLC stage C (53.8%), with 7 (53.8%) having received prior liver directed therapy. There were 5 patients (38.4%) having received 1 prior line of systemic therapy, and 8 with two prior lines of therapy. All received atezolizumab/bevacizumab as first line therapy. Macrovascular invasion was present in 4 (30.8%) patients. Complete baseline characteristics are presented in Table 1.

**Table 1.** Patient demographics and disease characteristics.

	Total (*n* = 13)
**Age**	
** Mean (SD)**	71.3 (7.61)
** Median (IQR)**	72.0 (64.0, 76.0)
** Range**	60.0, 87.0
** Missing**	0
**Gender, *n* (%)**	
** Female**	6 (46.2%)
** Male**	7 (53.8%)
**Race, *n* (%)**	
** White**	13 (100.0%)
**Ethnicity, *n* (%)**	
** Not Hispanic or Latino**	13 (100.0%)
**Prior lines of treatment, *n* (%)**	
** 1**	5 (38.4%)
** 2**	8 (61.5%)
**Macrovascular invasion, *n* (%)**	
** Yes**	4 (30.8%)
** No**	9 (69.2%)
**Multifocal disease, *n* (%)**	
** Yes**	6 (46.2%)
** No**	5 (38.5%)
** Unknown**	2 (15.4%)
**Ascites, *n* (%)**	
** Yes**	2 (15.4%)
** No**	10 (76.9%)
** Unknown**	1 (7.7%)
**Etiology, *n* (%)**	
** Alcohol Alone**	3 (23.1%)
** Multiple**	3 (23.1%)
** None/Unknown**	6 (46.2%)
** Viral Alone**	1 (7.7%)
**Child-Pugh Classification, *n* (%)**	
** Grade A**	12 (92.3%)
** Grade B**	1 (7.7%)
**BCLC, *n* (%)**	
** A**	1 (7.7%)
** B**	5 (38.5%)
** C**	7 (53.8%)
**Prior liver directed therapy, *n* (%)**	
** Yes**	7 (53.8%)
** No**	6 (46.2%)
**ECOG, *n* (%)**	
** 0**	5 (38.5%)
** 1**	8 (61.5%)
**Baseline Alpha Fetoprotein (µg/L), *n* (%)**	
** Mean (SD)**	613.0 (1334.10)
** Median (IQR)**	70.0 (23.0, 293.0)
** Range**	0.9, 4621.0
** Missing**	0
**Baseline Alpha Fetoprotein L3% (%), *n* (%)**	
** Mean (SD)**	47.0 (31.49)
** Median (IQR)**	55.0 (29.0, 69.0)
** Range**	1.0, 86.0
** Missing**	4
**Prior systemic regimen**	
** First line: Atezolizumab/bevacizumab**	13
** Second line: lenvatinib/sorafenib**	4
** Second line: durvalumab/tremelimumab**	2
** Second line: clinical trial**	2

The median duration of treatment was 9.9 weeks ([IQR]: 5.9-16.6) with median 3 (range 1.0-8.0) cycles of treatment (Tables S1-S3). The primary reason for discontinuation was radiological or clinical disease progression (8 [61.5%]) (Table S4).

### Efficacy

**Figure 1.**
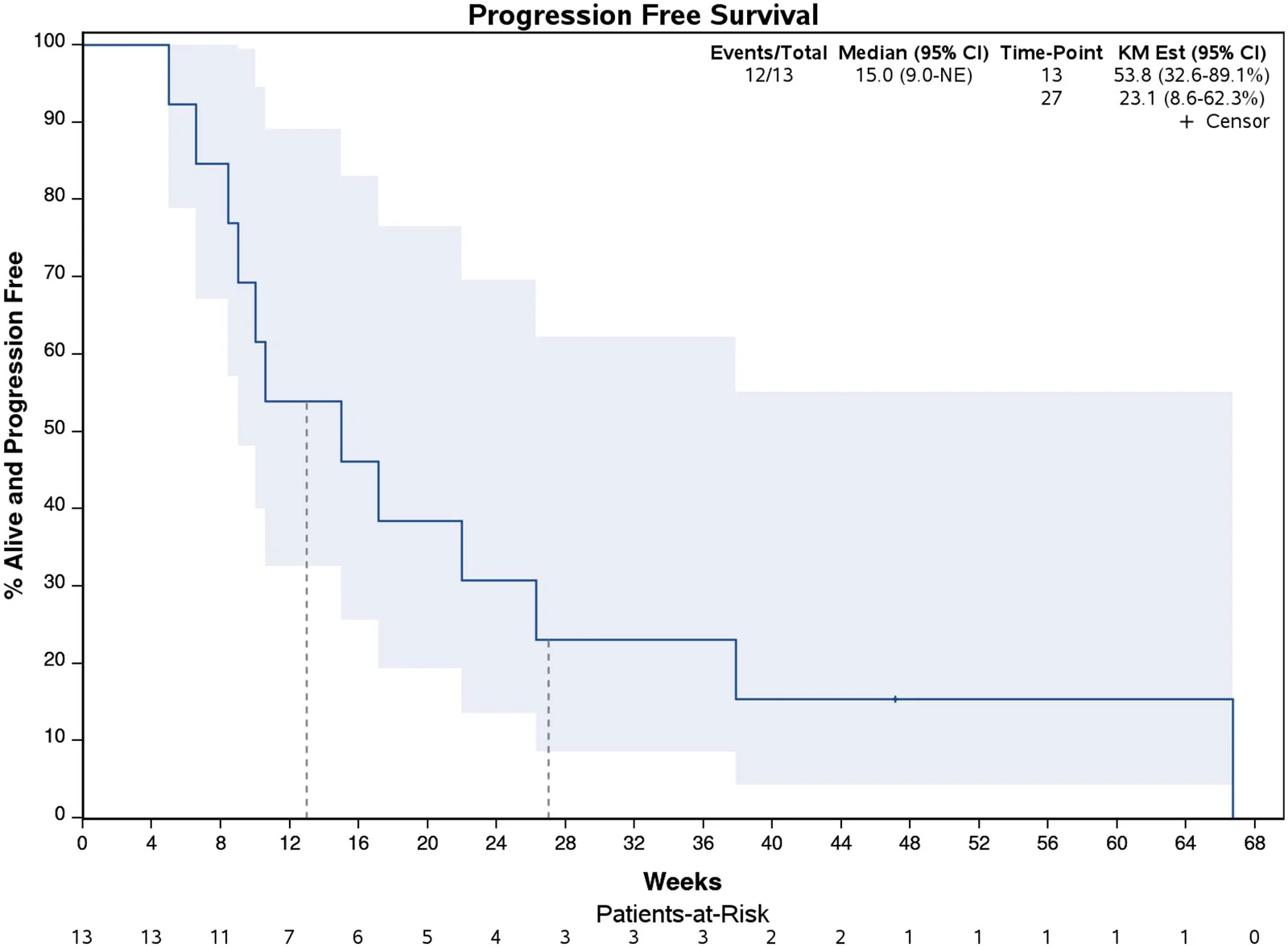
Primary endpoint, PFS at 6 months (ie, 27 weeks).

**Figure 2.**
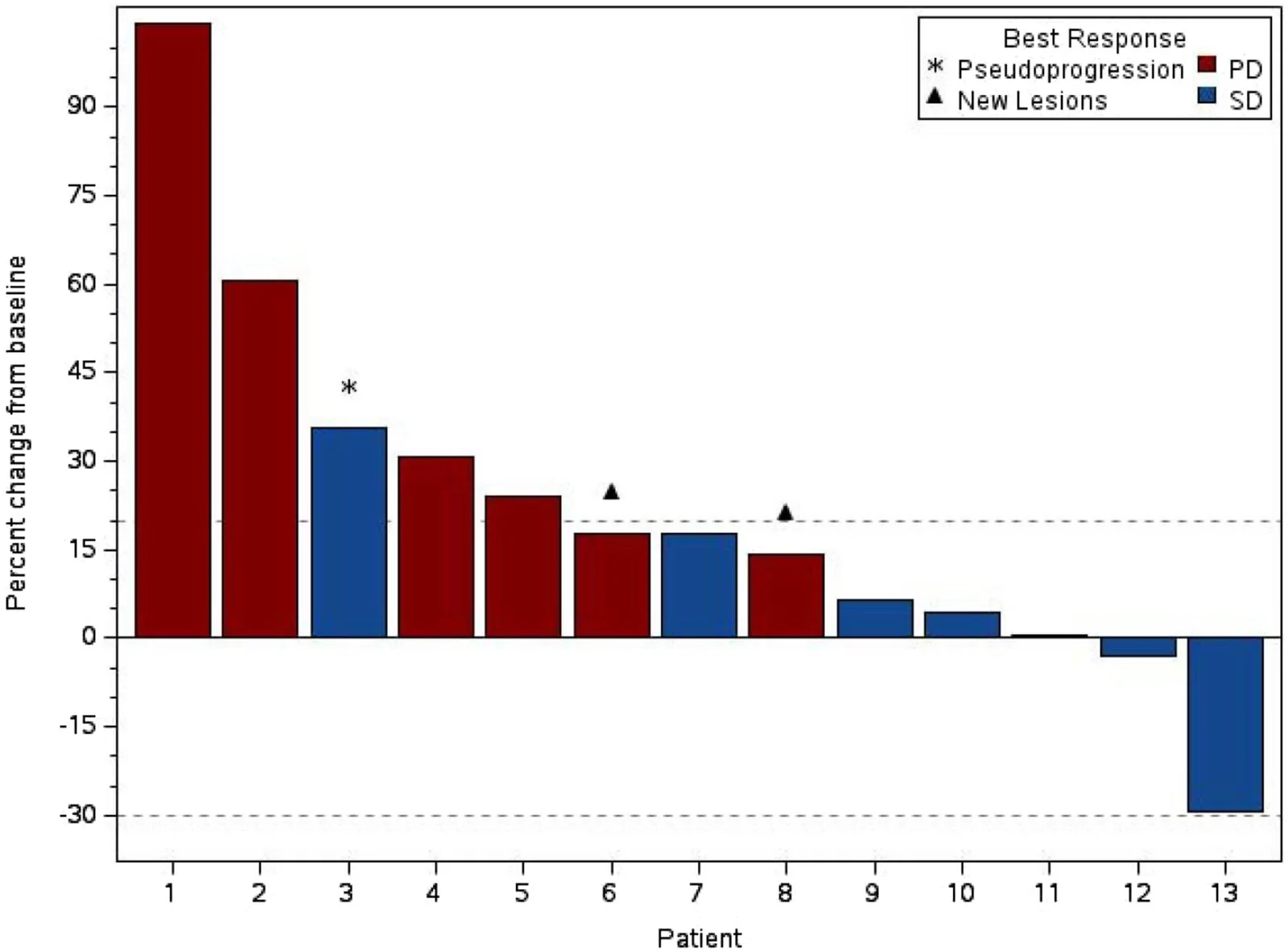
Waterfall Plot of Best Response.

**Figure 3.**
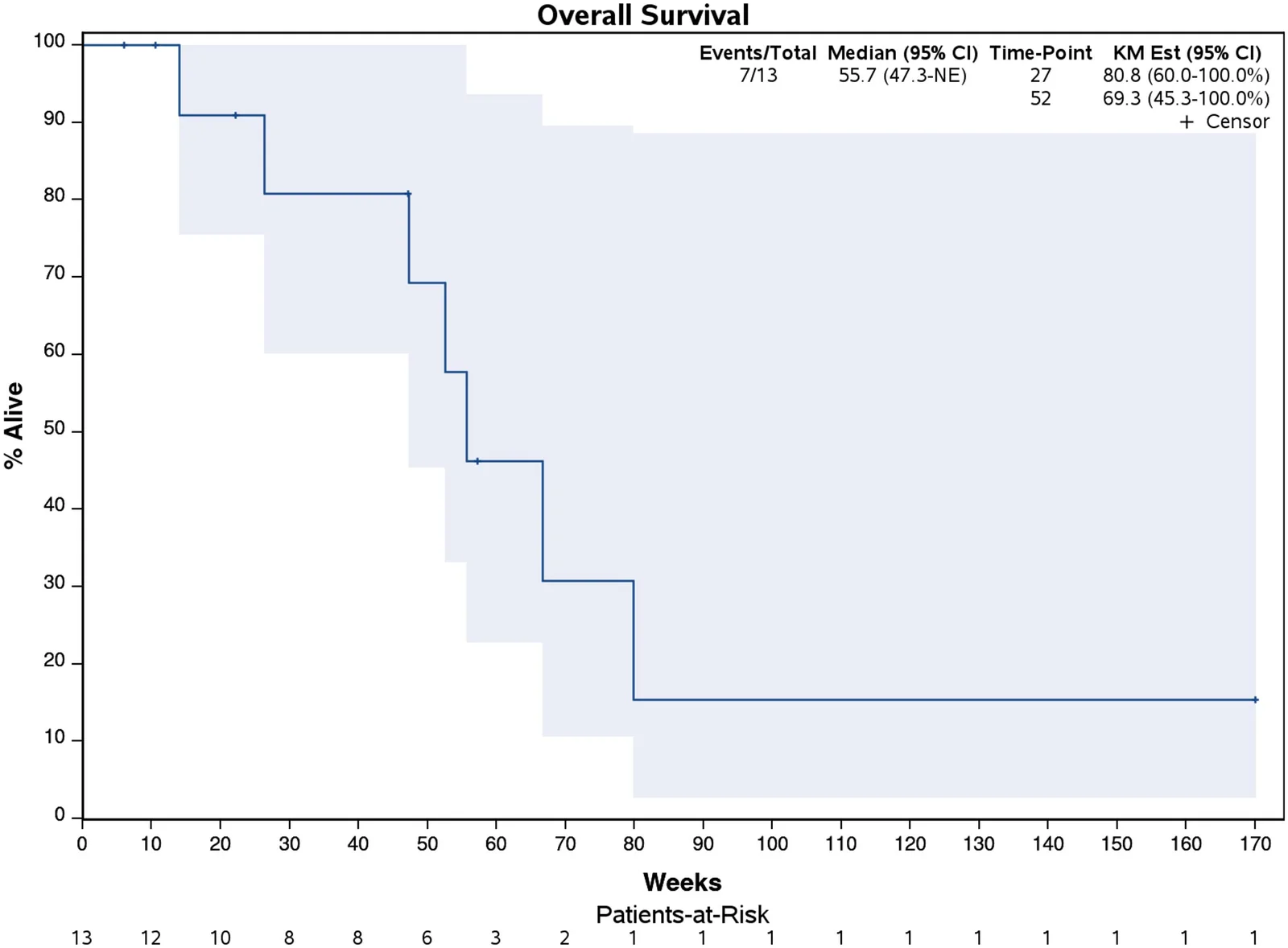
KM curve of overall survival.

Median PFS was 15 weeks (95% confidence interval [CI], 9.0-Not Estimable [NE]) and PFS rate at 6 months was 23.1% (95% CI, 8.6%, 62.3%) (Figure 1). Objective response rate was 0% (95% CI, 0%, 24.71%) in this study. Stable disease was observed in 7 (53.9%) patients (Table 2, Figure 2). With median follow-up of 57.3 weeks (IQR: 22.1-190.3), median overall survival was 55.7 weeks (95% CI, 47.3-NE) (Figure 3).

**Table 2.** Objective Response Rate.

Objective response	Total
**CR/PR**	0
**SD**	7 (53.85%)
**PD**	6 (46.15%)
**Total**	13

ORR (95% CI by Clopper-Pearson) is 0% (0%, 24.71%).

### Safety

Nine (69.2%) patients experienced at least 1 grade 3+ adverse events (AE) and two with grade 4 (myocarditis, tumor hemorrhage), regardless of attribution. The same patient with myocarditis had acute kidney failure grade 5 AE considered by clinical treating team as unrelated to study treatment (acute kidney failure in the setting of myocarditis and a medical history of IgA nephropathy). Most commonly reported AEs (occurring in more than 35% of patients) were hyperphosphatemia (76.9%), fatigue (69.3%), diarrhea (61.6%), AST (53.9%) and ALT (46.2%) increased, dry eye (38.5%), nail changes (38.5%), rash maculo-papular (38.5%), and oral mucositis (38.5%) (Table 3).

**Table 3.** Adverse event summary. All patients who have initiated treatment will be considered evaluable for adverse event analyses. 13 patients are evaluable for adverse event analyses. Grade 5 AE- Patient ID R0002748, AE term: Acute kidney injury, attribution: Unrelated.

		n	%
**Patients with at least one:**	**Arm**		
**Grade 3+ Adverse Event**	**A**	9	69.2
**Grade 4+ Adverse Event**	**A**	2	15.4
**Grade 3+ Hem Adverse Event**	**A**	3	23.1
**Grade 4+ Hem Adverse Event**	**A**	0	0.0
**Grade 3+ Non-Hem Adverse Event**	**A**	9	69.2
**Grade 4+ Non-Hem Adverse Event**	**A**	2	15.4

The AE table below sorts the AE by prevalence of any grades.			

Adverse events		Grade									
		1		2		3		4		5	
		*n*	%	*n*	%	*n*	%	*n*	%	*n*	%
**Type**	**Arm**										
**Hyperphosphatemia**	**A**	2	15.4	7	53.8	1	7.7				
**Fatigue**	**A**	6	46.2	2	15.4	1	7.7				
**Diarrhea**	**A**	3	23.1	4	30.8	1	7.7				
**Aspartate aminotransferase increased**	**A**	6	46.2	1	7.7						
**Alanine aminotransferase increased**	**A**	6	46.2								
**Dry eye**	**A**	5	38.5								
**Mucositis oral**	**A**	3	23.1	1	7.7	1	7.7				
**Nail changes**	**A**	5	38.5								
**Rash maculo-papular**	**A**	5	38.5								
**Abdominal pain**	**A**	1	7.7	2	15.4	1	7.7				
**Hyponatremia**	**A**	2	15.4	1	7.7	1	7.7				
**Creatinine increased**	**A**	1	7.7	1	7.7	1	7.7				
**Hypertension**	**A**			2	15.4	1	7.7				
**Lymphocyte count decreased**	**A**	1	7.7	1	7.7	1	7.7				
**Nausea**	**A**	1	7.7	1	7.7	1	7.7				
**Platelet count decreased**	**A**	3	23.1								
**Alkaline phosphatase increased**	**A**	2	15.4								
**Alopecia**	**A**			2	15.4						
**Anemia**	**A**					2	15.4				
**Anorexia**	**A**	2	15.4								
**Blood bilirubin increased**	**A**	1	7.7	1	7.7						
**Blurred vision**	**A**	1	7.7	1	7.7						
**Constipation**	**A**	2	15.4								
**Edema limbs**	**A**	1	7.7			1	7.7				
**Hyperglycemia**	**A**	2	15.4								
**Hypophosphatemia**	**A**	2	15.4								
**Peripheral sensory neuropathy**	**A**	1	7.7	1	7.7						
**Activated partial thromboplastin time prolonged**	**A**	1	7.7								
**Acute kidney injury**	**A**									1	7.7
**Anxiety**	**A**	1	7.7								
**Arthralgia**	**A**			1	7.7						
**Arthritis**	**A**	1	7.7								
**Back pain**	**A**	1	7.7								
**Bacteremia**	**A**			1	7.7						
**Blood and lymphatic system disorders—Other, specify**	**A**	1	7.7								
**Blood bicarbonate decreased**	**A**	1	7.7								
**Chronic kidney disease**	**A**			1	7.7						
**Confusion**	**A**			1	7.7						
**Dizziness**	**A**	1	7.7								
**Dry mouth**	**A**	1	7.7								
**Dry skin**	**A**	1	7.7								
**Dysgeusia**	**A**	1	7.7								
**Dyspnea**	**A**					1	7.7				
**Eye pain**	**A**			1	7.7						
**Gastrointestinal disorders—Other, specify**	**A**					1	7.7				
**Hematoma**	**A**					1	7.7				
**Hypercalcemia**	**A**	1	7.7								
**Hypocalcemia**	**A**	1	7.7								
**Hypokalemia**	**A**					1	7.7				
**Hypotension**	**A**					1	7.7				
**Joint range of motion decreased**	**A**			1	7.7						
**Memory impairment**	**A**			1	7.7						
**Muscle weakness lower limb**	**A**			1	7.7						
**Myalgia**	**A**			1	7.7						
**Myocarditis**	**A**							1	7.7		
**Nail loss**	**A**	1	7.7								
**Pain in extremity**	**A**			1	7.7						
**Palmar-plantar erythrodysesthesia syndrome**	**A**			1	7.7						
**Pleural effusion**	**A**					1	7.7				
**Pruritus**	**A**	1	7.7								
**Renal calculi**	**A**					1	7.7				
**Sinus bradycardia**	**A**	1	7.7								
**Thyroid stimulating hormone increased**	**A**	1	7.7								
**Tumor hemorrhage**	**A**							1	7.7		
**Urinary retention**	**A**					1	7.7				
**Vomiting**	**A**					1	7.7				

### Quality of life questionnaire-exploratory

Thirteen patients completed the EORTC-QLQ-C30 at baseline and only 7 completed the questionnaire at first restaging. Median scores (range) were 79.9 (51.5-100) at baseline and 68.2 (46.0-90.3) at first restaging. Change from baseline score has median (range) of −10.68 (−27.78, −2.39), based on 7 patients (Table S5).

### Molecular data

Of the 14 patients enrolled in the study, 11 (78.5%) had molecular profile completed through Tempus or FoundationOne. None of these profiles demonstrated FGF19 amplification. The median TMB was 3.7 m/MB with all being microsatellite stable. The most common mutations were *TERT, RB1, TP53, WT1 and CTNNB1*.

## Discussion

In this phase 2 study, we evaluated the efficacy and safety of futibatinib, a pan-FGFR Inhibitor, combined with pembrolizumab, an anti-PD1 antibody, in patients with advanced HCC expressing FGF19 following first-line and second-line therapy. The primary endpoint—progression-free survival at 6 months—was not met at interim analysis, and coupled with slow accrual, led to early study closure. Notably, over 53% of patients achieved stable disease, and the median overall survival was approximately 12.8 months. The overall survival estimate should be interpreted with caution given the small sample size and events.

Elevated FGF19 expression, via selective FGFR4 binding, is associated with poor prognosis across multiple malignancies—including breast, pancreatic, non-small cell lung, and hepatocellular carcinoma.^10^ Prior preclinical and clinical evidence supports FGF19 as a driver gene in HCC, and ongoing research is advancing FGF19/FGFR4-targeted therapies in this disease.^18^^,^^23^^,^^24^

Kim et al. previously demonstrated the clinical safety and efficacy of fisogatinib, a selective FGFR4 inhibitor, in advanced pretreated HCC.^18^ Among FGF19-expressing IHC-positive patients, a 17% response rate and a median PFS of 3.3 months were observed, whereas FGF19 IHC-negative patients showed no response. All patients had prior sorafenib exposure. In our study, the cohort was older, had at least one if not two prior lines of systemic therapy, did not achieve an objective response, but exhibited a comparable median PFS exceeding 3 months. One explanation for the lack of objective response is likely related to the inability of achieving sustained pathway inhibition, rather than selectivity. The investigational agent, a potent pan-FGFR inhibitor, forms covalent bond with a conserved cysteine residue in the ATP-binding pocket of the FGFR kinase domain, leading to inhibition of the kinase activity. Intrinsic potency has been established.^25–27^ However, to achieve/maintain a high enough concentration for sustained inhibition against FGFR4 protein, which has a rapid synthesis and turnover rate of 1-2 hours,^26^ maximum dose is needed. Our population had a difficult time with the full dosing schedule due to side effects and thus median dose density was 80%. This is further supported by Tao and colleagues in their assessment of pan-FGFR inhibition (FGFR3/FGFR4 expression in HCC) is superior to that of FGFR4-selective inhibitors.^28^

Of the 45 patients prescreened, 14 (31%) met the inclusion criteria, including FGF19 IHC-positivity—a rate slightly higher than previously reported.^18^^,^^29^ Notably, among those who had molecular profile completed (*N* = 11), none demonstrated *FGF19 locus* amplification, suggesting that epigenetic mechanisms may underlie majority of the increased expression. Though the small sample size limits this interpretation. Prior next generation sequening data indicated only 5%-10% of HCC tumor harbor *FGF19 locus* amplication.^30^ The absence of clinical response may reflect heterogeneity in FGF19 expression across tumor lesions, indicating that a 1% IHC threshold may not be sufficient to predict therapeutic response. Thus, a higher-stringent FGF19 scoring/threshold is required. Other stringent criteria may include H score quantification rather than binary cutoffs, mandatory multi-regional core biopsies to combat spatial heterogeneity, and incorporating quantitative RNA sequencing alongside IHC to establish transcript expression cutoff, while balancing costs and patient burden.

In this study, a significant proportion experienced grade 3 (9 [69.2%]) and grade 4 + (2 [15.4%]) adverse events. Most of these, as expected, were related to pan-FGFR inhibition and included hyperphosphatemia, dry eye, fatigue, diarrhea and nail changes. Elevated liver enzymes and rash were also observed likely related to pembrolizumab. One patient developed tumor hemorrhage grade 4 and another patient developed myocarditis grade 4. Tumor hemorrhage can occur spontaneously in HCC with incidence ranging from 3%-15%.^31^^,^^32^ Myocarditis is a known rare side effect of immunotherapy.^33^ The patient developed myocarditis first, leading to acute kidney failure in the setting of having a history of IgA nephropathy. The patient opted for hospice and subsequently passed away during the same hospitalization.

Second-line and later therapeutic options for advanced hepatocellular carcinoma remain notably limited, particularly in light of recent approvals for novel immunotherapy-based regimens. This study, along with others in the field, highlights several important lessons and challenges that have shaped the current landscape of HCC treatment.

One of the most significant barriers encountered was slow patient accrual, a challenge exacerbated by the COVID-19 pandemic. The pandemic not only disrupted clinical trial operations but also contributed to delays in patient recruitment and protocol adherence. Additionally, the presence of competing liver diseases among the patient population further complicated enrollment and treatment. Many patients who received immunotherapy-based first line regimen, particularly those with second line therapy as well, developed worsening liver function and thus were not eligible, which in turn reduced the pool of candidates eligible for subsequent line of interventions. This underscores the evolving nature of HCC management, where improvements in first-line options are reshaping expectations and strategies for subsequent lines of therapy.

Several limitations should be emphasized. The study was substantially underpowered, enrolling only 13 evaluable patients rather than the planned 22, and was stopped early because of futility and slow accrual; therefore, efficacy estimates, including PFS and OS, should be interpreted cautiously. In addition, only a small proportion of patients completed quality-of-life assessments at first restaging, raising the possibility of attrition bias and limiting conclusions regarding patient-reported outcomes. Future studies should prospectively incorporate strategies to improve quality-of-life data capture and should better characterize patients who derive prolonged disease control, including clinical features, treatment exposure, dose density, tumor biology, and biomarker patterns. Such analyses may help identify subgroups more likely to benefit and inform more refined biomarker-driven trial designs in the post-immunotherapy setting.

## Conclusion

In conclusion, this study demonstrated that conducting a biomarker selected clinical trial, particularly focusing on FGF19 expression, FGF19 ≥ 1% by IHC/ISH does not enrich sufficiently for benefit from futibatinib + pembrolizumab in previously treated aHCC; future efforts should focus on biomarker stringency and FGFR4-selective strategies and/or randomized post-immunotherapy designs. Novel agents are urgently needed. Identifying an optimal second-line or third-line regimen remains an important unmet need.

## Supplementary Material

oyag343_Supplementary_Data

## Data Availability

The data that support the findings of this study are available from the corresponding author, NHT, upon reasonable request.
