# Supplementary material for: Phase 2 study of futibatinib in combination with pembrolizumab in patients with FGF19 expressing advanced hepatocellular carcinoma
Source: Oncologist. 2026 Aug 25;31(10):oyag343. doi: 10.1093/oncolo/oyag343 (PMC13596051; doi:10.1093/oncolo/oyag343)
Supplement: Supplementary file 1 [file oyag343_supplementary_data.zip › Figure S1 Consort Diagram.docx]

**Figure S1 Consort Diagram**

Evaluable for Analysis (eligible, consented, FGF19+, received any protocol therapy)

N= 13

All patients registered

N= 14

First patient in: 08/17/2021 Last patient in: 03/18/2024

Patients not evaluable

N= 1

Reasons: Withdrawal before beginning protocol treatment
