# Supplementary material for: Phase 2 study of futibatinib in combination with pembrolizumab in patients with FGF19 expressing advanced hepatocellular carcinoma
Source: Oncologist. 2026 Aug 25;31(10):oyag343. doi: 10.1093/oncolo/oyag343 (PMC13596051; doi:10.1093/oncolo/oyag343)
Supplement: Supplementary file 1 [file oyag343_supplementary_data.zip › Table S1 Dosing Table.docx]

**Table S1 Dosing Table**

|  | Total (N=13) |
| --- | --- |
| Duration of Treatment, weeks |  |
| Mean (SD) | 11.8 (7.69) |
| Median (Q1, Q3) | 9.9 (5.9, 16.6) |
| Range | 1.4, 24.9 |
| N miss | 0 |
| Number of Treatment Cycles |  |
| Mean (SD) | 4.1 (2.43) |
| Median (Q1, Q3) | 3.0 (2.0, 6.0) |
| Range | 1.0, 8.0 |
| N miss | 0 |
