# Supplementary material for: Phase 2 study of futibatinib in combination with pembrolizumab in patients with FGF19 expressing advanced hepatocellular carcinoma
Source: Oncologist. 2026 Aug 25;31(10):oyag343. doi: 10.1093/oncolo/oyag343 (PMC13596051; doi:10.1093/oncolo/oyag343)
Supplement: Supplementary file 1 [file oyag343_supplementary_data.zip › Table S2 Dosing Table.docx]

**Table S2 Dosing Table: Futibatinib**

|  | Total (N=13) |
| --- | --- |
| Percent Targeted Dose |  |
| Mean (SD) | 80.1 (16.66) |
| Median (Q1, Q3) | 80.8 (63.5, 94.8) |
| Range | 52.4, 100.0 |
| N miss | 0 |
| At Least 1 Dose Delay |  |
| No | 9 (69.2%) |
| Yes | 4 (30.8%) |
| At Least 1 Dose Omission |  |
| No | 2 (15.4%) |
| Yes | 11 (84.6%) |
| # Dose Omissions |  |
| Mean (SD) | 13.8 (14.88) |
| Median (Q1, Q3) | 10.0 (2.0, 19.0) |
| Range | 0.0, 51.0 |
| N miss | 0 |
