# Supplementary material for: Phase 2 study of futibatinib in combination with pembrolizumab in patients with FGF19 expressing advanced hepatocellular carcinoma
Source: Oncologist. 2026 Aug 25;31(10):oyag343. doi: 10.1093/oncolo/oyag343 (PMC13596051; doi:10.1093/oncolo/oyag343)
Supplement: Supplementary file 1 [file oyag343_supplementary_data.zip › Table S3 Dosing Table.docx]

**Table S3 Dosing Table: Pembrolizumab**

|  | Total (N=13) |
| --- | --- |
| Percent Targeted Dose |  |
| Mean (SD) | 100.0 (0.00) |
| Median (Q1, Q3) | 100.0 (100.0, 100.0) |
| Range | 100.0, 100.0 |
| N miss | 0 |
| At Least 1 Dose Delay |  |
| No | 10 (76.9%) |
| Yes | 3 (23.1%) |
| At Least 1 Dose Omission |  |
| No | 13 (100.0%) |
| Yes | 0 (0%) |
| # Dose Omissions |  |
| Mean (SD) | 0.0 (0.00) |
| Median (Q1, Q3) | 0.0 (0.0, 0.0) |
| Range | 0.0, 0.0 |
| N miss | 0 |
