# Supplementary material for: Phase 2 study of futibatinib in combination with pembrolizumab in patients with FGF19 expressing advanced hepatocellular carcinoma
Source: Oncologist. 2026 Aug 25;31(10):oyag343. doi: 10.1093/oncolo/oyag343 (PMC13596051; doi:10.1093/oncolo/oyag343)
Supplement: Supplementary file 1 [file oyag343_supplementary_data.zip › Table S4 Off treatment reason.docx]

| Table S4 Off treatment reason | |
| --- | --- |
|  | Total (N=13) |
| **Off Treatment Reason**, n (%) |  |
| Adverse Event | 2 (15.4%) |
| hospice | 1 (7.7%) |
| Disease Progression During Protocol Intervention | 8 (61.5%) |
| Other Complicating Disease | 1 (7.7%) |
| Patient Withdrawal/Refusal After Beginning Protocol Intervention | 1 (7.7%) |
|  | |
