# Supplementary material for: Phase 2 study of futibatinib in combination with pembrolizumab in patients with FGF19 expressing advanced hepatocellular carcinoma
Source: Oncologist. 2026 Aug 25;31(10):oyag343. doi: 10.1093/oncolo/oyag343 (PMC13596051; doi:10.1093/oncolo/oyag343)
Supplement: Supplementary file 1 [file oyag343_supplementary_data.zip › Table S5 EORTC.docx]

**Table S5 EORTC-QLQ-C30 Change from Baseline to First Restaging (median [range])**

- A higher score indicates a better quality of life.
- A 10-point threshold has been used to assist regulators and health authorities interpret the meaningfulness of within-patient score changes (Ref: Osoba D, Rodrigues G, Myles J, Zee B, Pater J. Interpreting the significance of changes in health-related quality-of-life scores. J Clin Oncol. 1998;16(1):139–144.)

| **Baseline (N=13)** | **First Restaging (N=7)** | **Change from Baseline (N=7)** |
| --- | --- | --- |
| 79.9 (51.5, 100) | 68.2 (46.0, 90.3) | -10.68 (-27.78, -2.39) |
